# Supplementary material for: Complexome profiling on the Chlamydomonas lpa2 mutant reveals insights into PSII biogenesis and new PSII associated proteins
Source: J Exp Bot. 2021 Aug 26;73(1):245–62. doi: 10.1093/jxb/erab390 (PMC8730698; doi:10.1093/jxb/erab390)
Supplement: erab390_suppl_Supplementary_Dataset_S1 [file erab390_suppl_supplementary_dataset_s1.zip › Supplemental Dataset 1 - Excel List and all profiles/plots/PRPL13_Cre06.g264350.html]

Trivial name: PRPL13  
  
Euclidean distance: 2618.92  
Mean Intensity (WT): 597.32  
Mean Intensity (Mut): 490.89  
Distance: 4.38  
  
MapMan: protein.synthesis.ribosomal protein.prokaryotic.non-organellar.50S subunit;protein.synthesis.ribosomal protein.prokaryotic.unknown organellar.50S subunit.L13;protein.synthesis.ribosomal protein.prokaryotic.chloroplast.50S subunit.L13  
  
p value of intensity sums Welch test: 0.7150
